# A Case of a Chronic Pancreatic Pseudocyst Causing Atraumatic Splenic Rupture without Evidence of Acute Pancreatitis

**DOI:** 10.1155/2016/2192943

**Published:** 2016-10-24

**Authors:** P. Moori, E. J. Nevins, T. Wright, C. Bromley, Y. Rado

**Affiliations:** ^1^University of Liverpool Medical School, Liverpool, UK; ^2^University Hospital Aintree, Liverpool, UK; ^3^Department of General Surgery, Chrissie Tomlinson Memorial Hospital, George Town, Grand Cayman, Cayman Islands; ^4^Department of Radiology, Chrissie Tomlinson Memorial Hospital, George Town, Grand Cayman, Cayman Islands

## Abstract

Atraumatic splenic rupture is a rare complication of a pancreatic pseudocyst (PP), described in the setting of chronic pancreatitis. There is common understanding, within the literature, that an inflammatory process at the tail of the pancreas may disrupt the spleen and result in such splenic complications. The authors present a case report of a 29-year-old male with a PP, associated with chronic pancreatitis. The patient had a history of excessive alcohol intake and presented to the emergency department with a short history of abdominal pain and vomiting. He denied any significant history of trauma and serum amylase levels were normal. An admission computed tomography (CT) scan of the abdomen confirmed the presence of a PP in direct contact with the spleen. The CT also demonstrated a heterogenous hypodense area of the splenic hilum, along with perisplenic fluid. The patient was admitted for observation. His abdominal pain progressed, and he became haemodynamically unstable. An emergency ultrasound scan (USS) at this time revealed intra-abdominal haemorrhage. A subsequent CT confirmed splenic rupture, which was managed surgically with a full recovery. Few such cases are documented within the literature and more understanding of preempting such events is needed.

## 1. Introduction

Splenic rupture is a relatively common and well-documented phenomenon, typically following substantial blunt abdominal injury [1]. There are several cases in the literature documenting cases of atraumatic splenic rupture (ASR), but it remains an uncommon phenomenon and for this reason this diagnosis is often overlooked in a patient without a history of trauma [1–3]. ASR, although rare, is a life threatening process, which can result as a complication of chronic pancreatitis [2]. A recent systematic review of 845 cases of ASR reported a mortality of 12 percent [2].

Pancreatic pseudocysts (PPs) are found in 20–40% of patients with chronic pancreatitis [4, 5]. PPs are encapsulated, well-defined collections of pancreatic fluid, typically found in the lesser sac. There are various associated complications described with PPs, including rupture of the PP into the peritoneal cavity, infection, haemorrhage, or fistulation with a nearby organ. Involvement of the spleen with a PP is rare, with fewer than 50 reported cases amongst the English literature [6–19]. Here, we present a case of PP secondary to chronic alcoholic pancreatitis, complicated by ASR. The low occurrence and poor understanding of such cases make this a vital topic for awareness for emergency general surgeons.

## 2. Case Presentation

A 29-year-old male, with a history of excessive alcohol intake and chronic pancreatitis, presented to the emergency department at The Chrissie Tomlinson Memorial Hospital, Cayman Islands. He exhibited a 24-hour history of abdominal pain, which was increasing in intensity, without a history of significant trauma. He also had associated vomiting. An abdominal examination revealed upper abdominal tenderness but no signs of peritonism.

**Figure 1:**
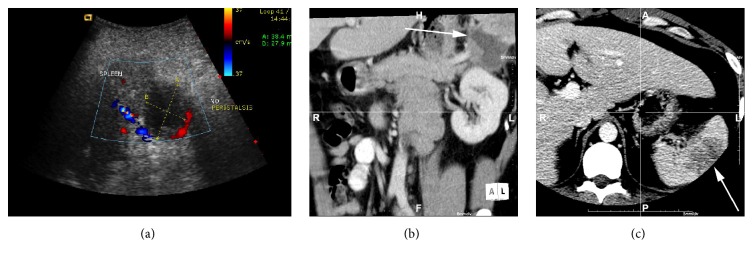
(a) Ultrasound scan demonstrating a pancreatic pseudocyst adjacent to the splenic hilum. (b) Coronal oblique reconstruction of contrast enhanced computed tomography. The arrow indicates the pancreatic tail pseudocyst, located at the splenic hilum. (c) Contrast enhanced computed tomography showing a heterogenous hypodense area in the spleen adjacent to the pancreatic pseudocyst. The white arrow refers to the spleen with hypodense area.

The patient was investigated with ultrasound scan (USS) that revealed a PP (Figure 1(a)). A computed tomography (CT) scan of the abdomen confirmed the presence of a PP in direct contact with the inferior pole of the spleen (Figure 1(b)). The patient was not known to have a PP prior to admission. A heterogenous hypodense area of the splenic hilum was noted and there was a grossly enlarged liver with a normal gall bladder (Figure 1(c)). Fat stranding was identified at the tip of the pancreatic tail with a thickened lateral conal fascia. A small fluid collection was seen between the upper pole of the spleen and the left liver. His serial serum amylase throughout this period remained within normal range, making acute pancreatitis less unlikely [20].

**Figure 2:**
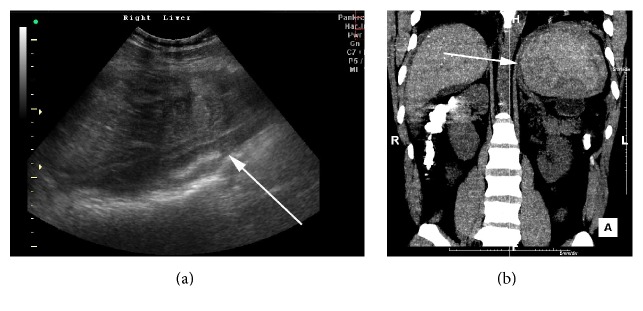
(a) USS showing a heterogenous liver and the presence of haemorrhagic fluid. (b) Computed tomography coronal reconstruction showing haemorrhage around the ruptured spleen. The arrow in (a) refers to haemorrhagic fluid in the liver and the arrow in (b) refers to the spleen.

Over the following 24 hours, the patient became haemodynamically unstable and showed signs of peritonism. An USS revealed intra-abdominal haemorrhage (Figure 2(a)), which was confirmed by CT as a result of a splenic rupture (Figure 2(b)). The patient was transfused and underwent an emergency laparotomy with splenectomy and distal pancreatectomy. The spleen was completely disintegrated and the parenchyma had shelled out of the capsule. The tail of the pancreas was densely adherent to the hilum of the spleen. The histology report did not demonstrate acute pancreatitis but identified variable inflammation, reactive changes, and focal necrosis, consistent with chronic fibrosing pancreatitis. Following surgery, the patient went on to make a full recovery. We believe that the PP directly contributed to atraumatic splenic rupture within this individual (ASR).

## 3. Discussion

Chronic pancreatitis, as in this case, can be complicated by the development of a PP in 20–40% of patients [4, 5]. A PP is usually located outside the pancreas and is a well-defined encapsulated fluid collection. The pathogenesis of PPs is considered to be due to the disruption of the pancreatic duct, due to increased ductal pressure [21]. 70–80% of PPs will spontaneously regress; however, some may persist and are usually monitored [4].

Rupture of the spleen is a well-documented event but is most commonly associated with blunt abdominal trauma [1]. However, cases of splenic rupture due to nontraumatic causes have been reported in patients with chronic pancreatitis, with 65 cases reported in the literature [22].

The pancreas and the spleen are anatomical allies and for this reason complications involving the spleen can occur as a result of pancreatitis or PP. Involvement of the pancreatic tail via pseudocysts or necrotizing pancreatitis has previously been shown to predispose patients to complications involving the spleen, such as splenic or portal venous obstruction and subcapsular haematomas [6, 21, 23]. The pancreatic tail, splenic hilum, its associated vessels, and the peritoneum of the anterior pancreatic surface are vital in allowing leaked pancreatic enzymes into the splenic parenchyma. Therefore, pseudocysts within the pancreatic tail allow diffusion of proteolytic enzymes, which result in disruption of the splenic hilum [24]. Moreaux and Bismuth [25] and Warshaw et al. [18] have demonstrated that pancreatic pseudocysts can result in splenic penetration, associated with haemorrhage and mortality. Therefore, the authors suggest elective excision of such pseudocysts via splenectomy and distal pancreatectomy. The most consistent finding for splenic involvement is upper abdominal pain and a history of pancreatitis [26]. Alongside this, laboratory investigations attempting to diagnose PP, with or without splenic involvement, are often nondiscriminatory [26].

At presentation, the CT scan showed an area of concern within the spleen. Hypodense areas of the spleen are commonly encountered on abdominal CT images, but the nature of these can be difficult to interpret and should be correlated with clinical findings. The majority of such lesions are benign and do not require specific follow-up or management; however, some findings along with clinical circumstances may necessitate further consideration [27]. Various morphologies such as appearance of the lesion, its borders, attenuation, and calcifications help to differentiate hypodense areas [28, 29]. For example, wedge shaped hypodense areas can be indicative of splenic infarction with ill-defined edges at the early stages and well-defined borders at later stages. The development of secondary splenic rupture and ensuing haemorrhage signify latent complications secondary to splenic infarction, which may be avoided with adequate CT analysis [30]. The authors propose that hypodense splenic areas in a patient with significant abdominal pain could indicate imminent splenic rupture. However, further work is needed to investigate PP and potential signs indicative of ASR.

We have reported a case of PP directly contributing to ASR, with no evidence of trauma. There have been several mechanisms of ASR secondary to PP described in the literature. It is possible that they all contribute towards ASR, and they have been included below for consideration: (1) dissection of the retroperitoneal sheath comprising the splenic vessels, leading to significant haemorrhage from the splenic artery, due to its anatomical location [12], (2) dissemination of proteolytic pancreatic enzymes through the splenic parenchyma invading through the hilum or along the associated vessels, causing consequent disruption such as in subcapsular splenic hematoma [19], (3) pseudocyst formation within ectopic intrasplenic pancreatic tissue, due to the expansive effects and capabilities of PPs [6, 15, 18, 21, 31], (4) direct erosion of the PP through the splenic capsule from vascular invasion, rendering the spleen vulnerable to ASR [12], and (5) splenic vessel thrombosis with subsequent liquefaction of a splenic infarct [19].

## 4. Conclusion

ASR secondary to PP in the absence of acute pancreatitis is a rare complication. We have hypothesised the pathogenesis for this infrequent occurrence. ASR should be considered and carefully monitored in patients complaining of abdominal pain where imaging highlights a PP in direct contact with the spleen or in patients with known PP who develop haemodynamic instability. We postulate that the hypodense appearance of the spleen in this case was suggestive of imminent rupture. We hope that increasing the awareness of such phenomena will enable emergency departments to diagnose similar cases.
